# Supplementary figures and images for: Hedgehog signaling controls astral microtubules and mitotic spindle orientation in neural progenitors and iPSCs
Source: Front Cell Dev Biol. 2025 Jun 6;13:1582924. doi: 10.3389/fcell.2025.1582924 (PMC12179144; doi:10.3389/fcell.2025.1582924)

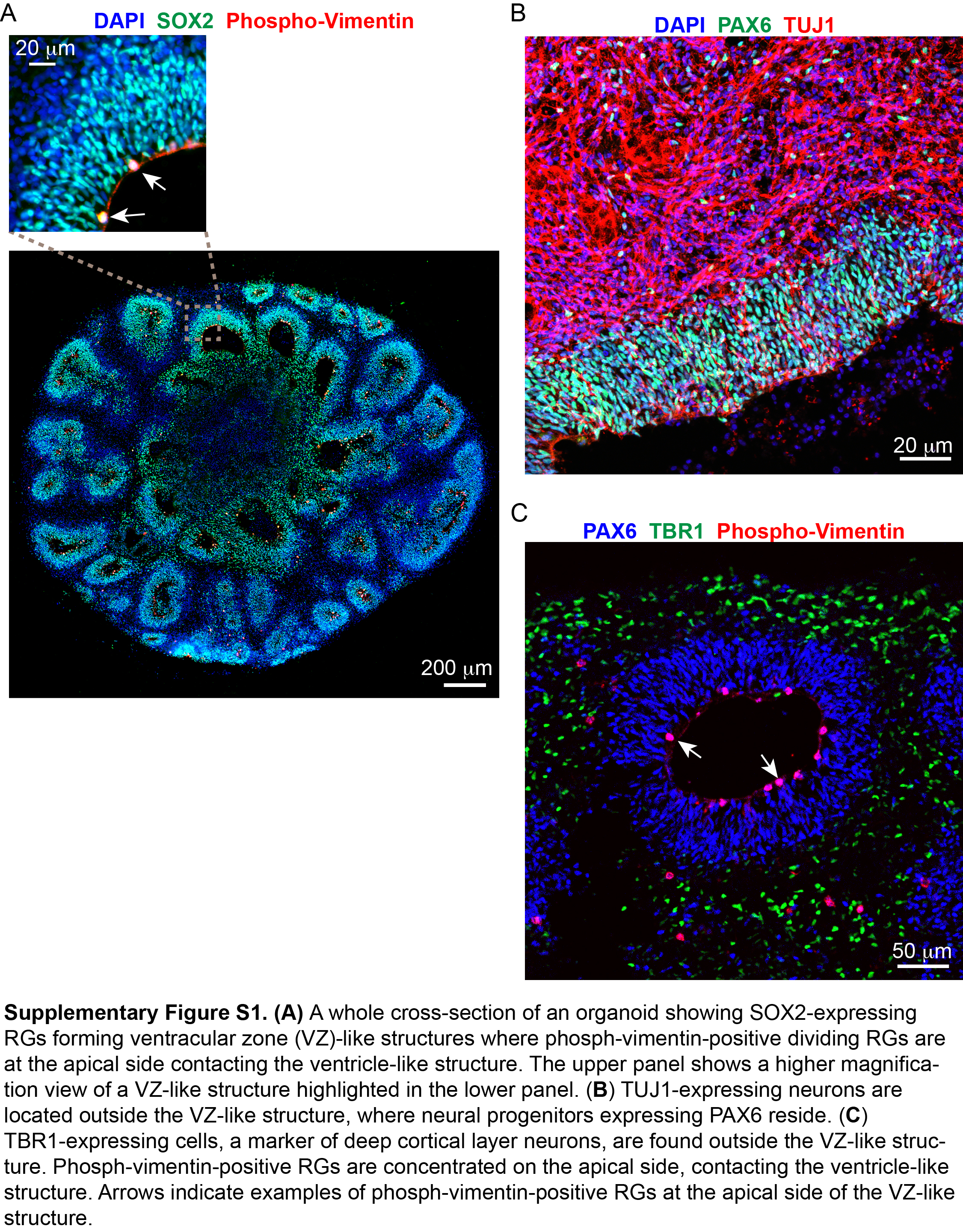

Supplement: Supplementary file 3 [file Image1.jpeg]

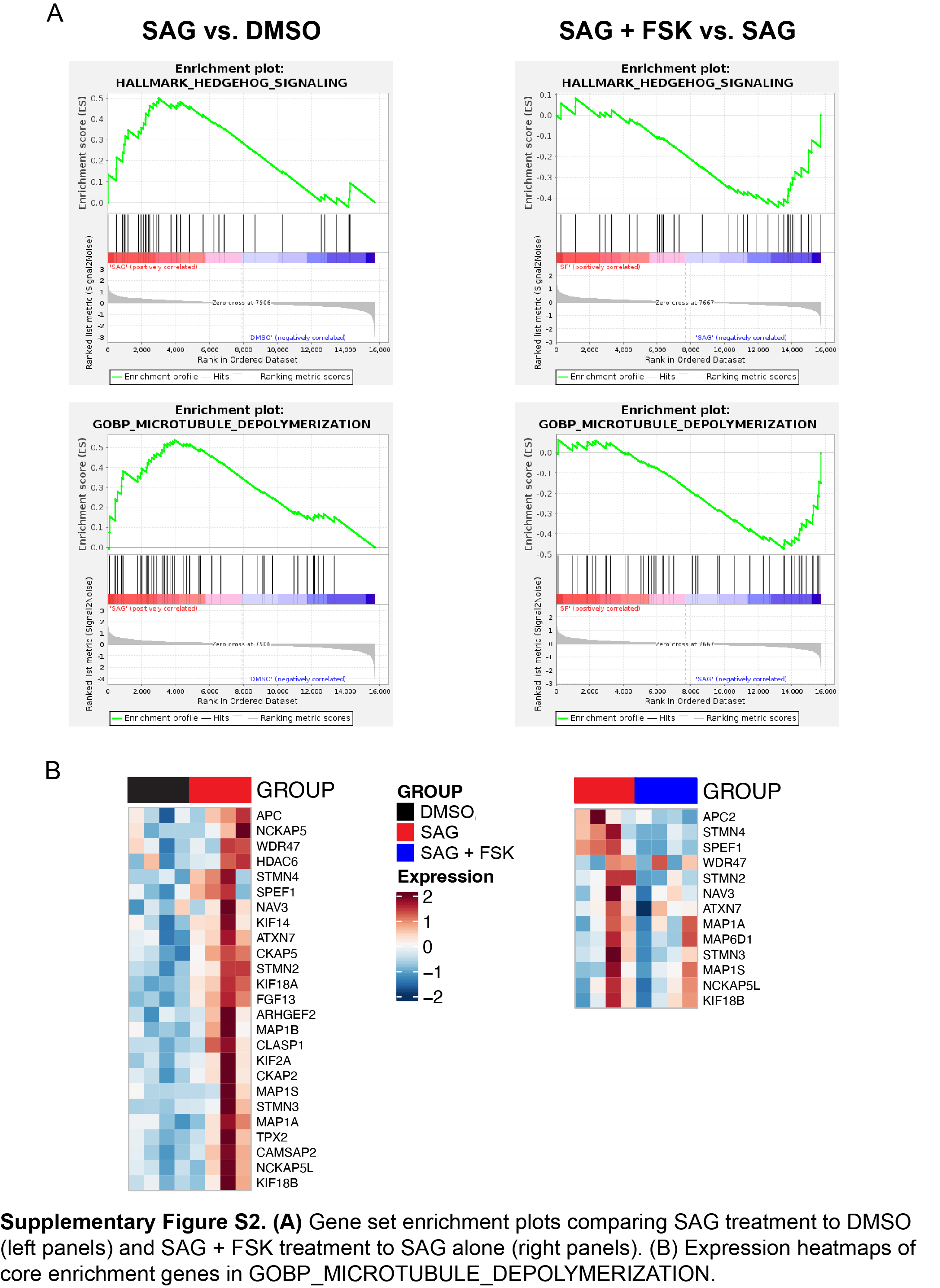

Supplement: Supplementary file 4 [file Image2.jpeg]
